# Cell Sheets Formation Enhances Therapeutic Effects of Human Umbilical Cord Mesenchymal Stem Cells on Spinal Cord Injury

**DOI:** 10.1111/cns.70163

**Published:** 2024-12-13

**Authors:** Yulin Zhao, Zhengchao Wu, Yuchen Zhou, Cheng Chen, Yang Lu, Heng Wang, Tao Xu, Changwei Yang, Xiaoqing Chen

**Affiliations:** ^1^ Department of Spine Surgery Affiliated Hospital of Nantong University Nantong China; ^2^ Medical School of Nantong University Nantong China

**Keywords:** angiogenesis, cell sheets, human umbilical cord mesenchymal stem cells, PI3K/Akt pathway, spinal cord injury

## Abstract

**Background:**

In recent years, the utilization of stem cell therapy and cell sheet technology has emerged as a promising approach for addressing spinal cord injury (SCI). However, the most appropriate cell type and mechanism of action remain unclear at this time. This study sought to develop an SCI rat model and evaluate the therapeutic effects of human umbilical cord mesenchymal stem cell (hUC‐MSC) sheets in this model. Furthermore, the mechanisms underlying the vascular repair effect of hUC‐MSC sheets following SCI were investigated.

**Methods:**

A temperature‐responsive cell culture method was employed for the preparation of hUC‐MSC sheets. The extracellular matrix (ECM) produced by hUC‐MSCs serves two distinct yet interrelated purposes. Firstly, it acts as a biologically active scaffold for transplanted cells, facilitating their attachment and proliferation. Secondly, it provides mechanical support and bridges spinal cord stumps, thereby facilitating the restoration of spinal cord function. The formation of the cavity within the spinal cord was evaluated using the Hematoxylin and Eosin (H&E) staining method. Subsequently, endothelial cells were cultivated with the conditioned medium (CM) obtained from hUC‐MSCs or hUC‐MSC sheets. The pro‐angiogenic impact of the conditioned medium of hUC‐MSCs (MSC‐CM) and the conditioned medium of hUC‐MSC sheets (CS‐CM) was evaluated through the utilization of the CCK‐8 assay, endothelial wound healing assay, and tube formation assay in an in vitro context. The development of glial scars, blood vessels, neurons, and axons in hUC‐MSCs and hUC‐MSC sheets was assessed through immunofluorescence staining.

**Results:**

In comparison to hUC‐MSCs, hUC‐MSC sheets demonstrated a more pronounced capacity to facilitate vascular formation and induce the regeneration of newborn neurons at the SCI site, while also reducing glial scar formation and significantly enhancing motor function in SCI rats. Notably, under identical conditions, the formation of cell sheets has been associated with a paracrine increase in the ability of the cells themselves to secrete pro‐angiogenic growth factors. During the course of the experiment, it was observed that the secretion of uPAR was the most pronounced among the pro‐angiogenic factors present in MSC‐CM and CS‐CM. This finding was subsequently corroborated in subsequent experiments, wherein uPAR was demonstrated to promote angiogenesis via the PI3K/Akt signaling pathway.

**Conclusion:**

The creation of cell sheets not only significantly enhances the biological function of hUC‐MSCs but also effectively retains the cells locally in spinal cord injury. Therefore, the transplantation of hUC‐MSC sheets can maximize the function of hUC‐MSCs, greatly reducing glial scar formation, enhancing vascular formation, and promoting the regeneration of neurons and axons. Additionally, the research findings prove that hUC‐MSC sheets activate the PI3K/Akt signaling pathway through uPAR secretion to enhance angiogenesis. The transfer of the entire extracellular matrix by hUC‐MSC sheets, in the absence of the introduction of additional exogenous or synthetic biomaterials, serves to further augment their potential for clinical application.

## Graphical abstract

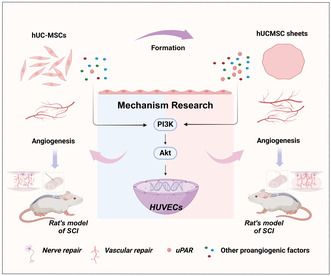

## 1 Introduction

Spinal cord injury (SCI) refers to the impairment of sensory and motor functions resulting from injury to spinal tissues, which can be permanent and irreversible [1, 2]. SCI is subdivided into two distinct phases: the primary and secondary injury phases [3, 4]. Primary injury is used to describe the initial impact on the spinal cord. In this stage, neural networks and the blood–brain barrier are damaged, which lead to interruptions in the blood supply. Secondary injury consists of a series of severe consequences that occur after the primary injury, such as extensive neuronal death, severe inflammatory reactions, and scar formation. Improving blood supply in SCI is considered a primary strategy for early intervention because ischemia can create an adverse microenvironment in the injury area. This strategy can promote axon regeneration, help clear inflammatory products, and provide nutritional support. Therefore, vascular regeneration should be promoted to accelerate neural recovery after SCI [5]. However, due to the persistent effects of inflammation, edema, and other secondary injuries, effective vascular formation in the injury center and its vicinity is difficult.

Using mesenchymal stem cells (MSCs) for vascular repair in SCI patients is a promising strategy. Recent studies indicate that the primary mechanism by virtue of which MSCs promote vascular formation SCI is through their paracrine effects. MSCs can produce beneficial cytokines, Extracellular vesicles (EVs), and other factors through paracrine secretion, thus promoting blood supply restoration in the injury area [6, 7, 8]. Most studies use methods such as intrathecal injection and tail vein injection [9, 10] for mesenchymal stem cell transplantation. These transplantation methods cannot make the cells act on the local injury of SCI, greatly weakening the function of MSCs. It is therefore imperative to guarantee the retention of stem cells at the site of SCI in order to ensure the efficacy of stem cell therapy.

Cell sheet technology is a well‐established technique, which was first invented by Okano et al. [11]. The preparation involves the use of a temperature‐responsive culture dish with a compound called poly‐N‐isopropylacrylamide (PIPAAm) conjugated to the bottom. The external temperature causes hydrophilic/hydrophobic changes in PIPAAm, resulting in thermoregulatory attachment and detachment of the cell culture dish and the cells. This temperature‐responsive culture dish can be recovered in the form of cells or cell sheets [12]. The advantage of this method is that it can extract all cells in a sheet form without disturbing the cell functions and the structure of the ECM, and without using any chemical or enzymatic treatment. Cell sheet technology overcomes the drawback of cell infusion not being able to have a long‐lasting effect in the injured area. Prior research has substantiated the viability of utilizing bone marrow mesenchymal stem cell (BMSC) sheets and adipose mesenchymal hematopoietic stem cell (ADSC) sheets for the management of SCI [13, 14, 15].

Currently, there are relevant studies in regard to the enhancement of nerve regeneration after SCI by cell sheets, but there is a paucity of research examining the impact on vascular endothelial cells and the spinal cord vascular system. Human umbilical cord mesenchymal stem cells (hUC‐MSCs) derived from neonatal umbilical cords can paracrine substances such as exosomes and growth factors that effectively promote vascular reconstruction [16, 17, 18], whose pro‐angiogenic effect is very significant [19], and have been proven that treating SCI is safe and beneficial [20, 21]. Therefore, we combined cell sheet technology for the first time, using hUC‐MSCs to prepare cell sheets, evaluated their safety and effectiveness, and used a rat SCI model to explore their angiogenesis and mechanism. We strive to use more methods and experiments to prove the possibility of hUC‐MSC sheets in the treatment of SCI.

## 2 Materials and Methods

### 2.1 Animal Preparation

The study population comprised 8‐week‐old female Sprague–Dawley (SD) rats. The rats were bred at the Experimental Animal Centre of Nantong University and had a mean body weight of approximately 220 g. The animals were provided with unrestricted access to standard rodent food and water and were housed in a controlled environment with a humidity of 50%, a temperature of 20°C–26°C, and a 12‐h light–dark cycle to ensure that the rats could move freely within the cage. All experimental procedures employed in this study were approved by the Animal Care and Use Committee of Nantong University and conducted in accordance with the National Research Council's Guide for the Care and Use of Laboratory Animals (No. S20240720‐002).

### 2.2 Isolation, Culture, and Identification of hUC‐MSCs

The hUC‐MSCs were obtained from the umbilical cords that excised during cesarean section. In brief, Wharton's jelly tissue was isolated from the umbilical cords and cultured in DMEM/F‐12 medium (Gibco) containing 10% FBS (Procell, Wuhan, China) and 1% penicillin–streptomycin. The cells were incubated at 37°C, 5% CO_2_, and appropriate humidity in an incubator. Once the density reached 70%–80%, the hUC‐MSCs were digested with trypsin–EDTA (0.25% (w/v), NCM Biotech). In order to ascertain the pluripotent differentiation capabilities of hUC‐MSCs, we employed the use of adipogenic, chondrogenic, and osteogenic induction differentiation assay kits. Flow cytometry (BD Biosciences FACSCalibur TM) was employed to ascertain the presence of specific surface antigens, including CD29, CD45, CD90, CD73, CD44, CD105, and HLA‐DR. The final quantification was conducted using a BD FACSAria II flow cytometer (BD Biosciences). The data were subsequently analyzed using flow cytometer data analysis software (FlowJo V10.8.1, FlowJo LLC, Ashland, OR, USA).

### 2.3 Preparation and Characterization of hUC‐MSC Sheets

To prepare hUC‐MSC sheets, the hUC‐MSC suspension was seeded at a seeding density of 1.3 × 10^5^ cells/cm^2^ on the surface of a 3.5‐cm temperature‐responsive cell culture dish (Thermo Fisher Scientific, Massachusetts, USA). When the cell confluence reached 90%–100%, vitamin C (50 μg/mL) was added to the culture medium to promote the production of ECM. The culture medium was replaced every 2 days and incubated in an incubator for 2 weeks. When it was time to collect, the incubation temperature was lowered to 20°C until the hUC‐MSC sheets detached from the bottom of the dish. The hUC‐MSCs would spontaneously form hUC‐MSC sheets, and complete hUC‐MSC sheets could be obtained subsequently by transferring the membrane. The structure of hUC‐MSC sheets was observed using an optical microscope, scanning electron microscope (SEM), and transmission electron microscope (TEM). Finally, we also performed immunofluorescence staining of fibronectin (Proteintech Group Inc., China) and 4′,6‐diamidino‐2‐phenylindole (DAPI) on frozen sections of hUC‐MSC sheets.

### 2.4 Collection of the Conditioned Media of hUC‐MSCs and hUC‐MSC Sheets

After the hUC‐MSCs formed sheets, they were divided into two groups on average. In one group, the cells on the sheet were redigested into a suspension using 0.25% EDTA–trypsin for cell counting, and an equal amount of P5 hUC‐MSCs was reseeded in a 3.5‐mm culture dish and cultured in DMEM/F12 medium containing 10% FBS and 1% penicillin–streptomycin to wait for the cells to reattach. In the other group, the cell sheets were placed in a 3.5‐mm culture dish and the same medium was added. After the cells were reattached, both groups were washed twice with phosphate‐buffered saline (PBS), replaced with DMEM/F12 medium for starvation treatment for 48 h to obtain the conditioned medium (CM), named MSC‐CM group and CS‐CM group, which were collected and stored in a 80°C refrigerator for further use. To explore the changes in the secretion of pro‐angiogenic factors by hUC‐MSCs before and after sheet formation, we used DMEM/F12 medium as the untreated control group.

### 2.5 Pro‐Angiogenic Growth Factor Assay

The growth factor array system (Human Angiogenesis Growth Factor Array; RayBiotech, Norcross, GA, USA) was used to semi‐quantitatively evaluate the pro‐angiogenic growth factors in the untreated control group, MSC‐CM, and CS‐CM. Each group was not diluted, and the number of cells was distributed according to the group. All procedures were performed in accordance with the user manual. ChemiDoc XRS (Bio‐Rad, Hercules, CA, USA) was used to image the chemiluminescence signals of the membrane spots, and the pro‐angiogenic growth factors in each group were quantified using Quantity software (Bio‐Rac). Each experimental group test was repeated at least three times. We performed statistical analysis of the results of duplicate tests with Image J software.

### 2.6 Western Blotting

Protein expression levels were assessed by Western blot analysis according to established standard protocols and instructions. The BCA protein kit (Beyotime) was used to determine the total protein concentration after cell lysis. Protein samples were then analyzed by sodium dodecyl sulfate ‐ polyacrylamide gel electrophoresis (SDS‐PAGE) and transferred to 0.45 μm pore size PVDF membranes (Millipore, USA). The PVDF membrane was then incubated in 5% BSA for 1 h to prevent non‐specific protein binding. Proteins were then separated by SDS‐PAGE and transferred to polyvinylidene difluoride membranes. The samples were then incubated with antibodies against PI3K (Proteintech), p‐PI3K (Abcam), Akt (Proteintech), p‐Akt (Proteintech), uPAR (Proteintech), and GAPDH (Abcam) for 16–18 h at 4°C. The samples were then washed with three‐phase buffered saline and Tween‐20 (TBST). The proteins were then incubated with goat anti‐rabbit IgG H&L (HRP) (Proteintech) for 2 h at room temperature. The bands were visualized and the target proteins quantified using enhanced chemiluminescence reagents (ECL, Beyoccl‐Plus, Abcam) and Image J software. All original blot images were available in Appendix S1.

### 2.7 CCK‐8 Proliferation Assay

The cultured HUVECs were digested, centrifuged, and then evenly divided into three groups. The supernatant was removed, and the untreated basal medium, MSC‐CM, and CS‐CM were respectively taken to uniformly resuspend the three groups of HUVECs. The grouped cell suspensions were seeded in a 96‐well plate (5000 cells per well) and incubated at 37°C and 5% carbon dioxide for 1 h until the cells adhere to the wall. Then, 10 μL of CCK‐8 solution was added to each well containing cells, and continued to incubate for 6 h (37°C, 5% carbon dioxide). After the incubation was completed, an enzyme‐linked immunosorbent assay reader (Termo Fisher) was used to test the absorbance of the plate at 450 nm every 1 h and the optical density (OD) value recorded.

### 2.8 Endothelial Cell Wound Healing (Cell Migration) Assay

The migration of endothelial cells on a 6‐well plate was determined by a scratch test. HUVECs were planted in a 6‐well plate (5 × 10^5^cells/well) and nurtured in DMEM/F12 medium containing 10% fetal bovine serum for 24 h to ensure cell viability, followed by starvation overnight in the medium without serum. At the bottom of each well, the adherent cells were radially centered using a 200 μL sterile pipette tip, then washed three times with PBS to remove the floating cells, and the scratch was observed in a timely manner under a microscope. Each well was treated with 2 mL of stored DMEM/F12 basal medium, MSC‐CM, or CS‐CM for HUVECs. To guarantee the precision of the results obtained for the rate of cell migration, the related factors with cell proliferation were excluded. At the same time, HUVECs were incubated in the presence of the anti‐mitotic agent mitomycin (Sigma Aldrich, USA). After 12 h, the wound was examined using an inverted phase‐contrast microscope. The resulting data were analyzed using Image J (v1.4, National Institutes of Health, Bethesda, Maryland) to distinguish the non‐healing area of the cells. Healing index = (initial area − remaining area)/initial area × 100%.

### 2.9 Tube Formation Assay

Matrigel (Corning) and DMEM/F12 basal medium were pre‐mixed on ice in a 1:1 ratio to produce a dilution. 200 μL of the Matrigel dilution was added to each well of a 24‐well plate and let stand for 1 h in a cell culture incubator, waiting until the Matrigel dilution is completely solidified. HUVECs were resuspended in DMEM/F12 basal medium, MSC‐CM, and CS‐CM, and 200 μL of the cell suspension (5 × 10^4^ cells/well) was added to each well of a 24‐well plate. Then, it was incubated for 6 h in a cell culture incubator at 37°C and the formation of cell tubes was observed under an inverted microscope. In the following experiments, in order to determine whether uPAR is involved in the PI3K/Akt pathway to promote endothelial cell tube formation, the same method was used. ImageJ software (National Institutes of Health, Bethesda) was used to calculate the number of meshes/junctions/nodes/branches of capillaries and tubular structures for quantification.

### 2.10 Construction of Lentivirus‐Transfected hUC‐MSCs Sheets and Collection of Conditioned Medium

hUC‐MSCs were seeded in 96‐well plates at a density of 10 × 10^4^ cells/mL, and 100 μL of cell suspension was added to each well. Lentiviruses carrying green, fluorescent uPAR RNA interference and non‐RNA‐interference lentiviruses (Genechem, Shanghai, CHINA) were retrieved from the −80°C refrigerator. An adequate quantity was used. the original virus solution was added and diluted with complete medium to titers of 1 × 10^7^ TU/mL, 5 × 10^7^ TU/mL, 1 × 10^8^ TU/mL, and 1.5 × 10^8^ TU/mL in four groups, corresponding to four different MOI gradients, 10, 50, 100, and 150, to determine the optimal multiplicity of infection conditions. Subsequently, different concentrations of puromycin were added to determine the lowest concentration that effectively killed the cells as the working concentration. The successfully constructed hUC‐MSCs were fabricated into cell sheets and divided into the uPAR RNA interference group (LV‐uPAR‐RNAi) and the non‐RNA‐interference group (LV‐GFP), and the collection of CM was similar to the aforementioned process.

### 2.11 Elisa

In accordance with the instructions provided by the manufacturer, the uPAR kit was used to determine the expression level of uPAR in the control group, LV‐GFP group, and LV‐uPAR RNAi group. Briefly, the collected Control, LV‐GFP‐CM, and LV‐uPAR RNAi CM samples were taken out from the −80°C freezer and centrifuged for 10 min at 3000 rpm. Standard, samples, and HRP‐labeled detection antibodies were appended to the wells, respectively, and hatched at 37°C for 1 h. Finally, TMB chromogenic reagent was added, and the stop solution was added after 15 min. The OD value at 450 nm was ascertained through the utilization of a microplate reader, with the expression level of the protein in the sample subsequently calculated in accordance with the standard curve.

### 2.12 EDU Proliferation Assay

The proliferation of HUVEC was measured using the Click‐iTTM EdU (Beyotime, Shanghai, CHINA) cell proliferation kit. First, HUVEC were resuspended in DMEM/F12 basal medium, LV‐GFP‐CM, and LV‐uPAR RNAi CM, seeded in 24‐well plates at a density of 2 × 10^5^ cells/mL, and incubated for 24 h. Then, the original medium was removed, and the medium containing 10 mM EdU was added and incubated for 1 h. The medium was removed after the completion of EdU‐labeled cells, and 4% paraformaldehyde (PFA) was used to stabilize the cells. The reaction solution was added in the dark according to the reagent instructions. The cell nuclei were stained with DAPI, observed, and photographed by a fluorescence microscope (1905Leica, USA). The number of EdU‐positive cells was determined using Image J. The cell proliferation rate was measured by dividing the number of EdU‐positive cells by the total number of cells.

### 2.13 Cell Invasion Assay

To determine whether uPAR was effective in the migration of endothelial cells mediated by the PI3K/Akt pathway, a Transwell migration chamber (Corning) was used to determine the chemotactic movement of HUVECs, and the groups were the same as the EDU experiment. Matrigel matrix gel and DMEM/F12 medium were diluted on ice at a ratio of 1:8. The Transwell insert was placed into a 24‐well plate, 100 μL of Matrigel matrix gel dilution was added to each well and placed in a 37°C incubator for 1 h to allow the gel to solidify completely. 600 μL of DMEM/F12 basal medium, LV‐GFP‐CM, and LV‐uPAR‐RNAi‐CM were added to each well in the lower chamber of the Transwell. HUVECs (cultured in DMEM/F12 medium containing 200 μmol/L for 12 h) were seeded at a density of 5 × 10^4^ cells/well in the upper chamber of the Transwell and 300 μL of cell suspension was added to each well. After 18 h, a sterile cotton swab was used to remove the non‐migrated cells on the surface of the upper chamber of the Transwell. Then, it was washed three times with 1 × PBS, fixed with 4% PFA for half an hour, and continued to wash three times with 1 × PBS. 500 μL of crystal violet solution (Byotime) was added to the lower chamber of the Transwell, stained for half an hour, and washed three times with 1 × PBS. After drying, a blank slide was placed on the microscope stage, and the upper chamber of the Transwell was placed upside down on the slide for photography.

### 2.14 Establishment of an SCI Model

hUC‐MSC sheets are harvested on the day of surgery and stored in specialized medium until transplantation. SD rats were anesthetized with 20 mg/kg sodium pentobarbital by intraperitoneal injection. Alamine resection is performed at the T10 stage, and then a suitable area of SCI is selected using a tailor‐made double row of scalpels (spaced 2 mm apart) to make a transverse cut from the middle to the left to create a uniform incision on the left side. The SD rats were randomly arranged into four groups: the sham operation group with only the vertebral plate removed, the SCI group with only injury without treatment, the MSC group with hUC‐MSCs implanted in the SCI group, and the CS group with hUC‐MSC tablets implanted based on the SCI group, with 15 SD rats in each group. The muscles, fascia, and epidermis were sutured sequentially, and the incision was disinfected with iodine. The rats were then injected with penicillin (40 × 10^5^ units/tablet/day, i.m.) and buprenorphine (0.01 mg/kg, i.m.). Our team manually emptied the bladder three times a day until the rat regained reflex control of the bladder.

### 2.15 H&E Staining and Preparation of Tissue Sections

One and 4 weeks after the operation, the required number of rats were euthanized, and perfusion fixation was performed using PBS and 4% PFA. The tissue sections of the lesion site was collected, incubated in 4% PFA for 1 day, and then gradient dehydration was performed using different concentrations of sucrose solution. The collected spinal cord tissue was cut to an appropriate length and embedded with OCT around it. Then, the tissue was placed in a freezing thermostat (Leica, Wetzlar, Germany) for operation to obtain coronal sections of about 8–10 μm thickness. Using hematoxylin and eosin (H&E) staining preparations (Boytime) as instructed, frozen sections were randomly selected from different groups and stained. After dehydration in an alcohol gradient and washing with xylene, it was sealed with neutral resin, and the staining was observed using an optical microscope (Leica).

### 2.16 Immunofluorescence Staining

The tissue sections were rewarmed at RT for 30 min. The sections were cleaned three times with PBS, incubated with 0.3% Triton X‐100 for 30 min, and then incubated with 10% normal goat serum at room temperature for 60 min. We added the primary antibody, and the mixture was incubated at 4°C for 16 h. The secondary antibody was then incubated in the same manner for 2 h, and the cell nuclei were stained with DAPI (Biosharp). All sections were observed and photographed using a Leica fully automatic inverted fluorescence microscope. For cell immunofluorescence, the cells were seeded on circular coverslips and fixed with 4% PFA, and the remaining procedures were similar to those for tissue sections. GFAP (Proteintech), CD31 (Santa Cruz Biotechnology, Dallas, TX, USA), NF200 (Abcam), Tuj‐1 (Abcam) were the primary antibodies.

### 2.17 Functional Recovery and Footprint Analysis

We assessed the recovery of hindlimb function in rats using the CatWalk XT system (Noldus Information Technology, Wageningen, the Netherlands). Briefly, paw prints were collected in the fourth postoperative week in rats, footprint strength was measured, and parameters were calculated using the software. To compare the difference between the injured side hindlimb and the normal hindlimb, we chose to use the ratio of the step length of the left hindlimb to that of the right hindlimb to determine the step length of the rats; the area of the left hind paw was divided by the area of the right hind paw to determine the print area. To exclude the interference of factors such as the body weight and paw size, the average strength was calculated by dividing the absolute value of the difference between the strength of the right hind paw and the strength of the left hind paw by the strength of the right hind paw. On Days 0, 1, 3, 7, 14, 21, and 28 after the rats underwent surgery, the rats were housed in the middle of a large open and closed field so that they could move freely. Three experimenters were selected for a double‐blind assessment to determine joint mobility, limb coordination, and trunk stability. The Basso, Beattie, and Bresnahan (BBB) scores were on an ordinal scale from 0 to 21.

### 2.18 Data Analysis

Each experimental project was repeated at least three times. Statistical analysis was carried out using GraphPad Prism 8.0.2 software (San Diego, USA), and the data of each group were presented in the form of mean ± SD. One‐way analysis of variance and Tukey's post hoc test were used to analyze the statistical differences between groups. The BBB score was analyzed by repeated two‐way analysis of variance and Tukey's post hoc test. When the *p* value was < 0.05, it was regarded as statistically significant.

## 3 Results

### 3.1 Identification of hUC‐MSCs and Preparation of hUC‐MSC Sheets

**FIGURE 1:**
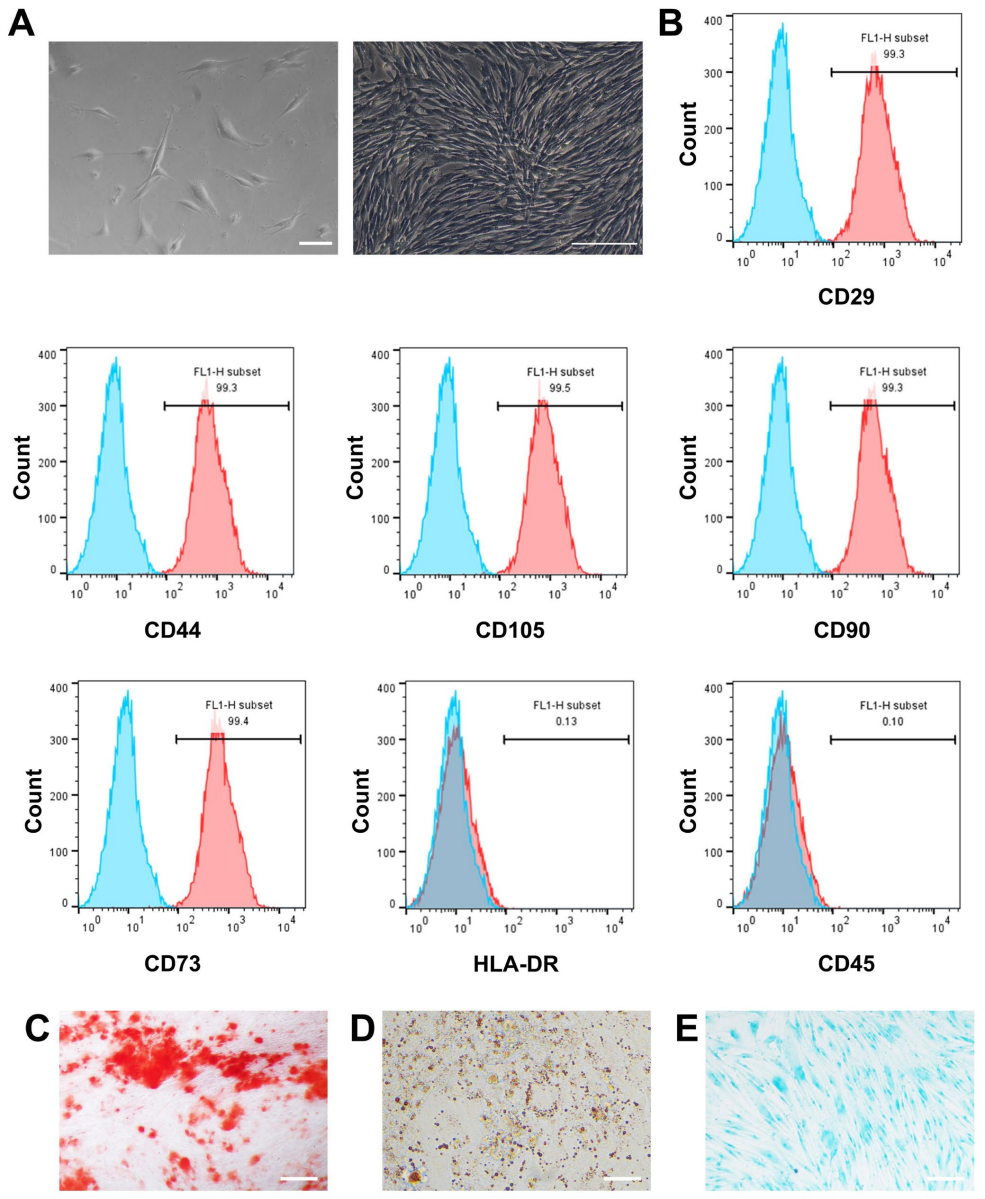
Morphology and characteristics of hUC‐MSCs. (A) Representative microscopic images of hUC‐MSCs. Fibroblast‐like morphology at low density (left), parallel or spiral morphology at high density (right). (B) Flow cytometry analysis of surface markers of hUC‐MSCs. (C–E) hUC‐MSCs show multipotent differentiation ability into osteogenic, adipogenic, and chondrogenic lineages. Scale bars: 100 μm (A); 200 μm (C, E); and 50 μm (D).

**FIGURE 2:**
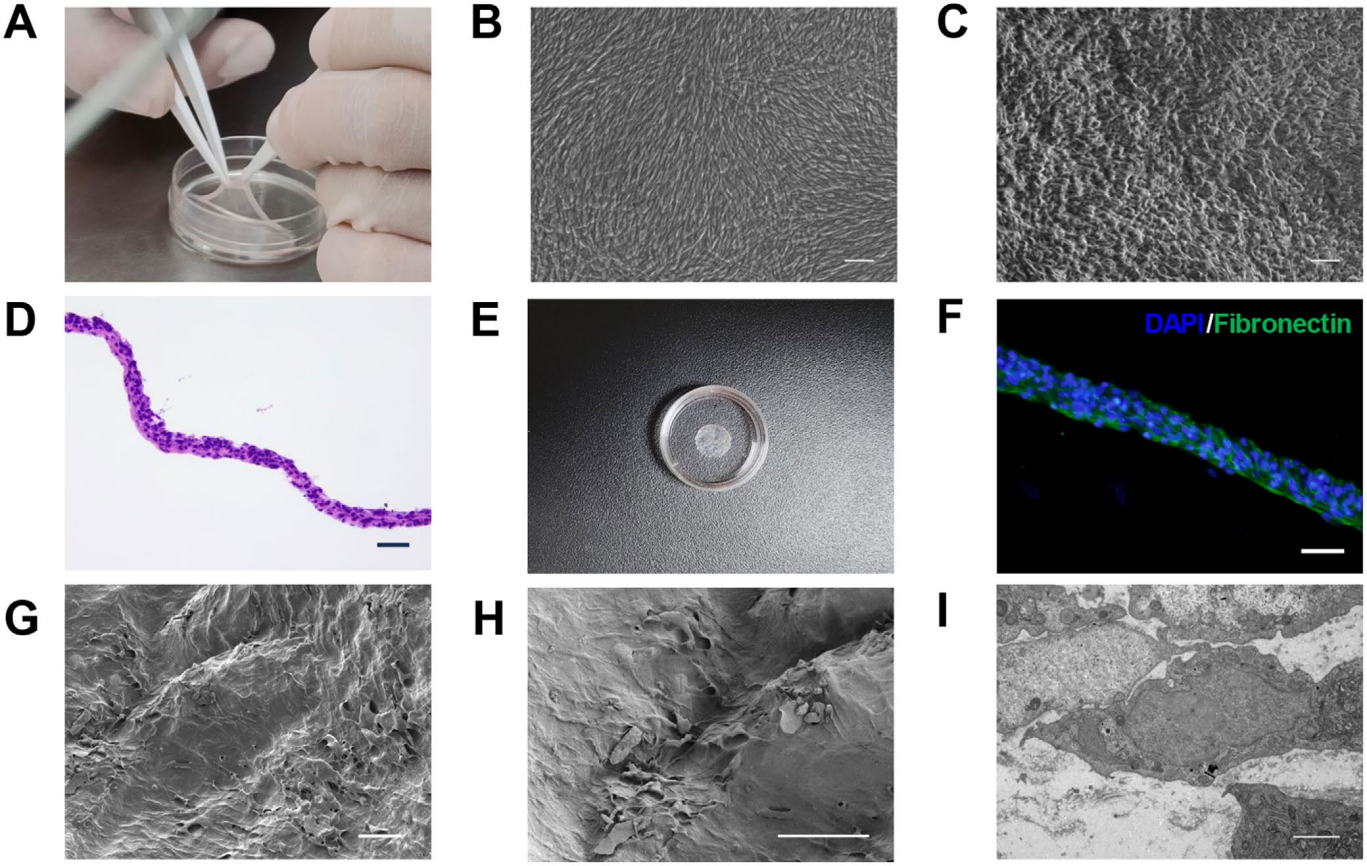
Preparation and characterization of hUC‐MSC sheets. (A) Acquisition of hUC‐MSC sheets. (B) Light microscopy observation of hUC‐MSC sheets not detached from the bottom of the culture dish. (C) Light microscopic observation of hUC‐MSC sheets detached from the bottom of the culture dish. (D) Histology of H&E of the cross section of the hUC‐MSC sheets. (E) hUC‐MSCs formed hUC‐MSC sheets after 14 days of culture. (F) Fluorescence microscopy view of the morphology of hUC‐MSC sheets. (G) Scanning electron microscopy of hUC‐MSC sheets. (H) Enlarged view of scanning electron microscopy of the hUC‐MSC sheets. (I) Transmission electron microscopy of hUC‐MSC sheets. Scale bars: 100 μm (B, C); 50 μm (D); 200 μm (F); 100 μm (G, H); and 2 μm (I).

In this study, we used hUC‐MSCs to prepare cell sheets. Observed under an inverted microscope, hUC‐MSCs adhered to the wall and showed a more typical fibroblast‐like spindle and polygonal cell morphology. At a higher density, the cells exhibited a relatively uniform spindle shape and grew in a parallel or spiral pattern (Figure 1A). Flow cytometry showed that hUC‐MSCs prominently expressed stem cell surface markers CD44, CD29, CD90, CD105, and CD73 (positive rate > 98%), but HLA‐DR and CD45 expressions were negative (positive rate < 1%; Figure 1B). The results of Oil red O, alizarin red S, and alcian blue staining assays showed that hUC‐MSCs can differentiate in multiple directions, such as chondrocytes, osteoblasts, and adipocytes (Figure 1C–E). After 14 days of induction with a medium containing vitamin C, hUC‐MSCs formed dense hUC‐MSC sheets and could detach from the bottom of the culture dish (Figure 2A). Inverted microscopy showed that the cell sheets maintained their original cell morphology before detachment (Figure 2B), and the hUC‐MSCs in the cell sheets were in a contracted state after detachment (Figure 2C). Moreover, the ECM also shrank due to its tension (Figure 2E). HE and immunofluorescence staining showed the positional relationship between the cell nucleus and the ECM (Figure 2D–F). Scanning electron microscopy showed that hUC‐MSC sheets were composed of multiple cell layers (Figure 2G,H). Transmission electron microscopy showed that hUC‐MSCs in the cell sheet were tightly connected, and there was abundant ECM between the cells (Figure 2I).

### 3.2 MSC‐CM and CS‐CM Contain Growth Factors Related to Angiogenesis

**FIGURE 3:**
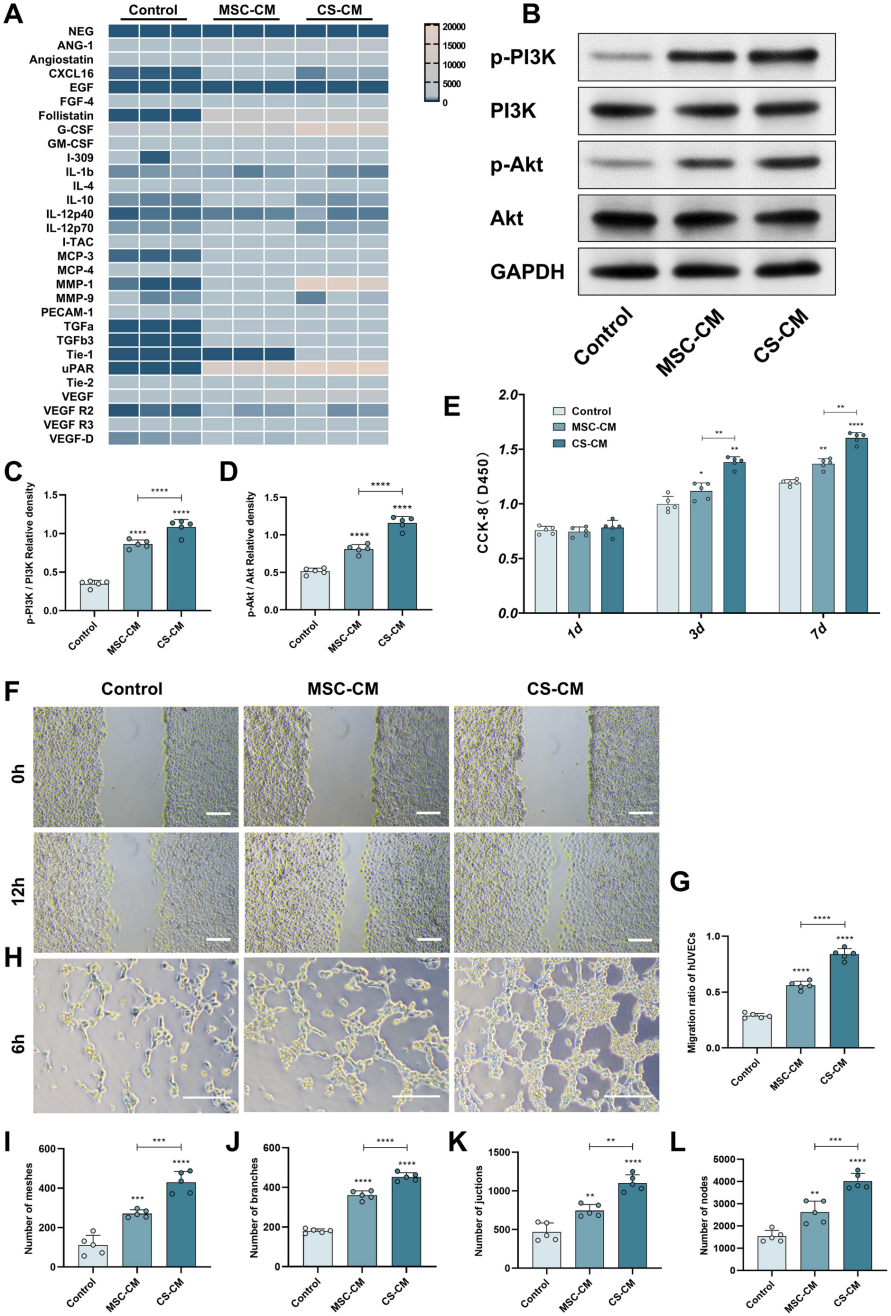
Mechanisms of the hUC‐MSC sheet in promoting angiogenesis. (A) Growth factor array of angiogenic factors. The color change of the heat map shows the quantitative changes of growth factors in MSC‐CM and CS‐CM or control medium. (B) Western blotting was used to detect the protein levels of p‐PI3K, PI3K, p‐Akt, and Akt in each group. (C, D) Relative densities of Akt and PI3K phosphorylation levels, respectively. (E) Eeffect of CS‐CM culture on the cell viability of CCK‐8 on HUVECs. (F) Phase‐contrast micrographs of HUVECs at the initial time and 12 h after monolayer scratch. (G) Migration of HUVECs in the scratch when using different medium groups. Healing index = (initial area − normal area)/initial area × 100%. (H) Representative images of in vitro HUVEC tube formation after culturing with MSC‐CM, CS‐CM, or the control medium. (I–L) Quantitative assessment of the number of capillary nodes, branches, connections, and meshes (rings) of HUVECs after treatment in each group. Scale bar: 100 μm (F, H). The data in panels (B–E, G, and I–L) passed the Shapiro–Wilk test and exhibit a Gaussian distribution. Data are presented as mean ± SEM (*n* = 5), *p < 0.05; **p < 0.01; ***p < 0.001; ****p < 0.0001; and ns, not significant.

The growth factors of MSC‐CM and CS‐CM were significantly higher than those of the control group. And the amounts of some factors were very significantly different from those of the control group, such as ANG‐1, G‐CSF, MMP‐1, uPAR, VEGF‐A, and Follistatin (Figure 3A). After hUC‐MSCs formed sheets, their secretion changed. Notably, among these measured pro‐angiogenic factors, the secretion of uPAR was the highest, and there was also a large difference in the amount of uPAR contained in CS‐CM and MSC‐CM.

### 3.3 MSC‐CM and CS‐CM Promote Angiogenesis Through the PI3K/Akt Signaling Pathway

Previous research results have shown that the activation of the PI3K/Akt pathway can promote the proliferation and migration of vascular endothelial cells [22, 23]. Additionally, uPAR, VEGF, etc. can mediate the activation of the PI3K/Akt pathway [24, 25]. The results indicated that the phosphorylation levels of Akt and PI3K in HUVECs in the CS‐CM group were higher than those in the MSC group and the control group. Moreover, the blot bands and gray value analysis demonstrated that compared to the Control group, the phosphorylation levels of PI3K and Akt in the MSC‐CM group and the CSM‐CM group changed significantly. Therefore, we speculate that in MSC‐CM and CS‐CM, uPAR might increase the phosphorylation levels of PI3K and Akt by activating the PI3K/Akt pathway (Figure 3B–D).

### 3.4 The Influence of MSC‐CM and CS‐CM on the Biological Activities of Vascular Endothelial Cells

The proliferation rate of HUVECs in the MSC‐CM group and the CS‐CM group increased. In the scratch test, the area of the CS‐CM group narrowed significantly. Compared with the control group, the MSC‐CM group also showed a significant narrowing trend, which means that some substances secreted by hUC‐MSCs and hUC‐MSC sheets can effectively benefit the migration of vascular endothelial cells. In the tube formation test, when HUVECs were co‐cultured with CS‐CM, a more typical tubular structure was formed on the matrix gel (Figure 3E–L).

### 3.5 hUC‐MSC Sheets Promote Angiogenesis by Secreting uPAR to Activate the PI3K/Akt Signaling Pathway

**FIGURE 4:**
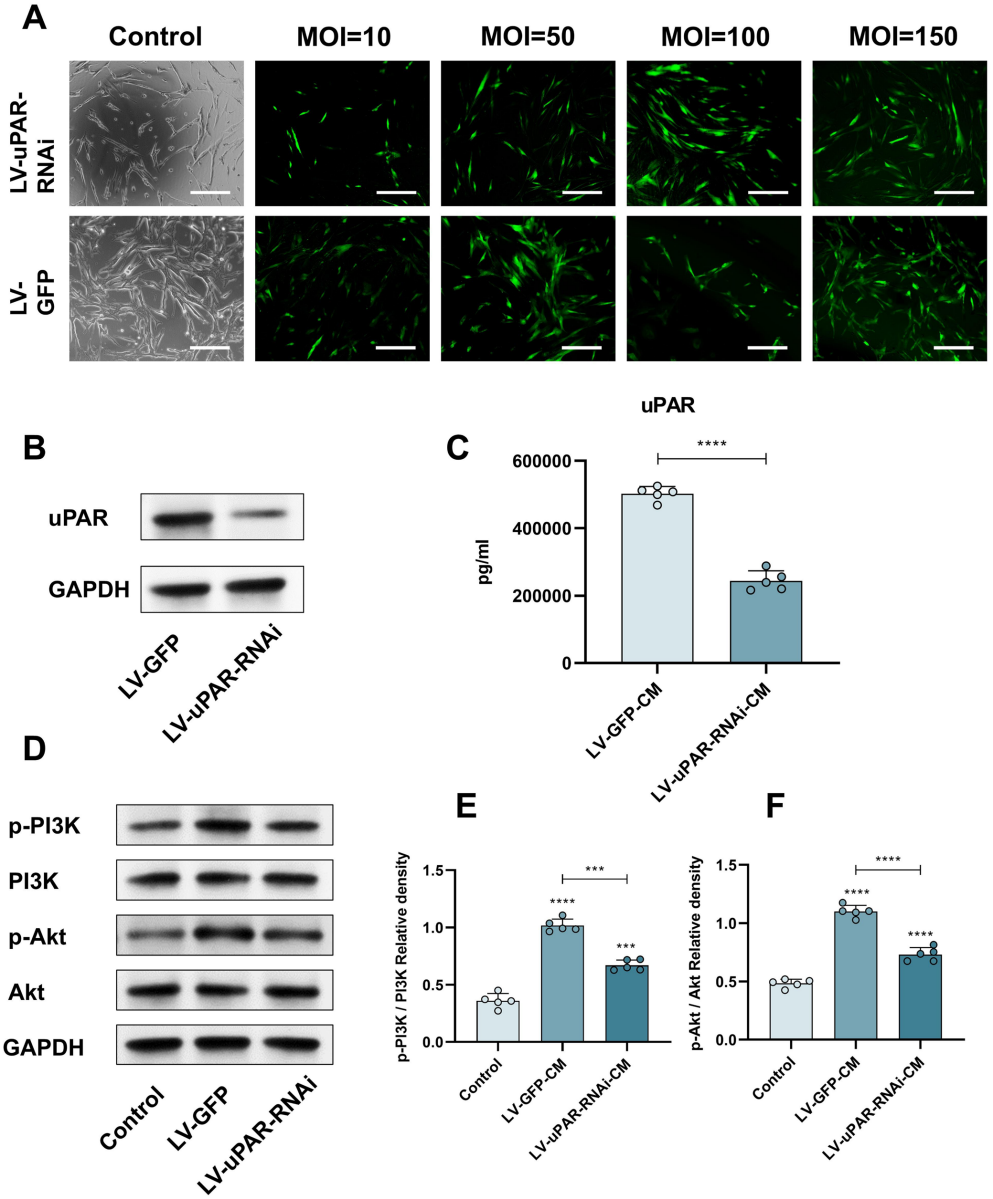
Lentivirus‐mediated uPAR interference reduces the secretion of uPAR and affects the activation of the PI3K/Akt pathway. (A) Screening for the optimal MOI for infecting hUC‐MSCs. (B) Detection of uPAR protein expression by Western blot. (C) Detection of uPAR secretion by Elisa. (D) Detection of the protein levels of p‐PI3K, PI3K, p‐Akt, and Akt in the Control group, LV‐GFP group, and LV‐uPAR group by Western blot. (E, F) Relative density of Akt and PI3K phosphorylation levels. Scale bar: 200 μm (A). The data in panels (C, E, and F) passed the Shapiro–Wilk test and exhibit a Gaussian distribution. Data are presented as mean ± SEM (*n* = 5), *p < 0.05; **p < 0.01; ***p < 0.001; ****p < 0.0001; and ns, not significant.

**FIGURE 5:**
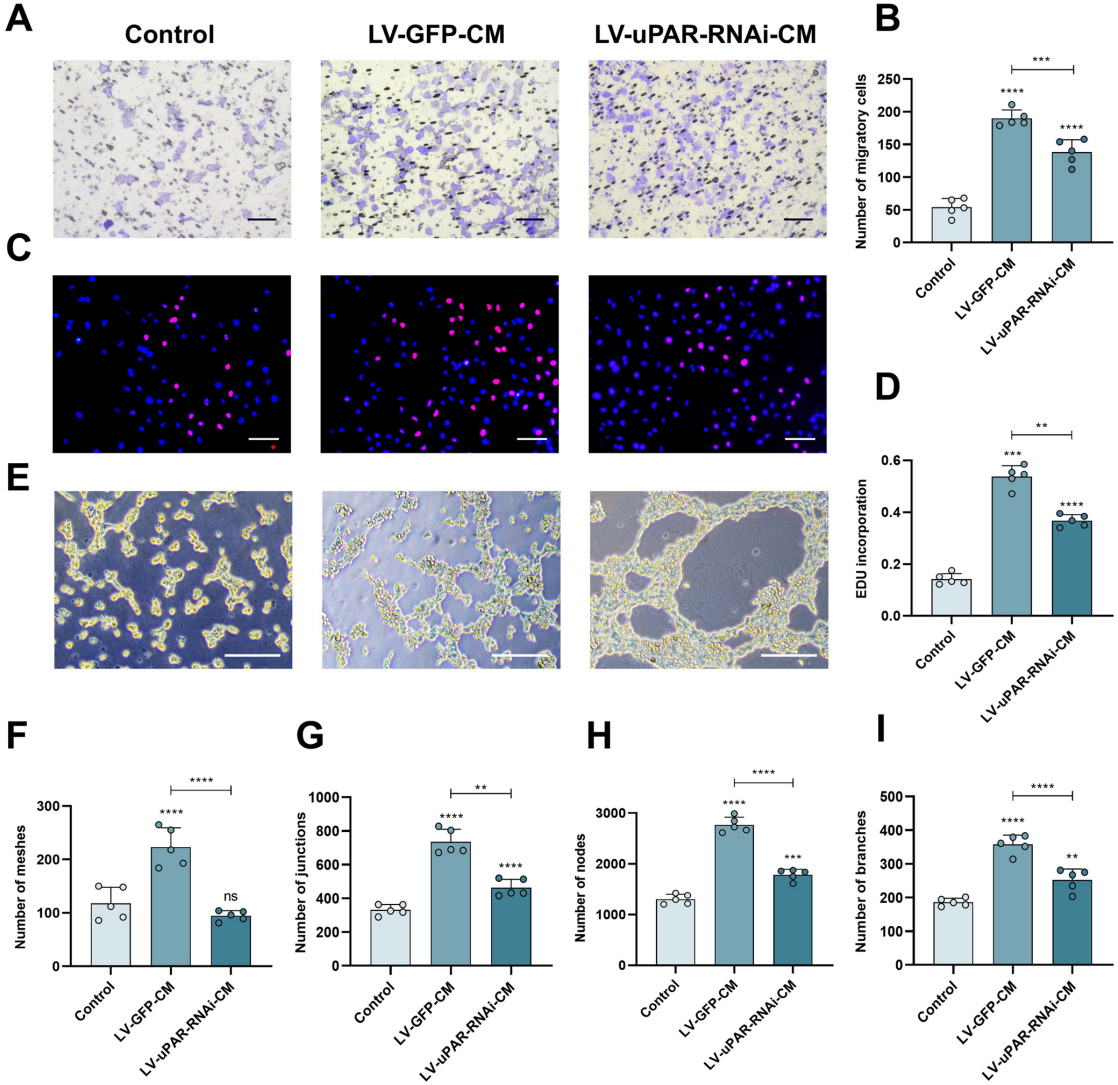
Effects of LV‐UPAR‐RNAi‐CM on HUVECs after uPAR interference. (A, B) Effects of LV‐GFP‐CM, LV‐UPAR‐RNAi‐CM, or control medium on the migration of HUVECs in Transwell migration chambers. (C, D) Effects of the Control group, LV‐GFP‐CM, and LV‐UPAR‐RNAi‐CM on the proliferation of HUVECs. (E) Representative images of in vitro HUVEC tube formation after culturing with LV‐GFP‐CM, LV‐UPAR‐RNAi‐CM, or control medium. (F–I) Quantitative evaluation of the number of capillary nodes, number of branches, number of connections, and number of meshes (rings) in HUVECs after treatment with each group. Scale bar: 100 μm (A, C, E). The data in panels (B, D, and F–I) passed the Shapiro–Wilk test and exhibit a Gaussian distribution. Data are presented as mean ± SEM (*n* = 5), *p < 0.05; **p < 0.01; ***p < 0.001; ****p < 0.0001; and ns, not significant.

Next, we investigated whether uPAR activates the PI3K/Akt pathway to exert a pro‐angiogenic effect. First, we performed lentiviral transfections on hUC‐MSCs to reduce their uPAR expression. Figure 4A shows that the lentivirus was successfully transfected into hUC‐MSCs, and the most suitable MOI value was selected. Then, Western blot analysis was used to detect a significant reduction in uPAR protein expression in the LV‐uPAR‐RNAi group compared to the LV‐GFP group (Figure 4B), and the actual secretion of uPAR in the LV‐GFP‐CM group and the LV‐uPAR‐RNAi‐CM group was measured using Elisa (Figure 4C). The results indicate that the lentivirus successfully performed uPAR RNA interference on hUC‐MSCs and reduced the secretion of hUC‐MSCs. To determine whether uPAR activates the PI3K/Akt pathway, we set up a Control group, an LV‐GFP‐CM group, and an LV‐uPAR‐RNAi‐CM group to culture HUVECs and detect the changes of PI3K and Akt proteins. The results show that the phosphorylation levels of PI3K and Akt in the LV‐uPAR‐RNAi group are decreased (Figure 4D–F). By seeding HUVECs in the upper chamber and the grouped media in the lower chamber, the motility of the cells was evaluated through a Transwell migration experiment. We observed that compared to the corresponding Control group, cell migration was significantly increased in both the LV‐GFP‐CM group and the LV‐uPAR‐RNAi‐CM group (Figure 5A,B). In addition, we also analyzed the proliferation of HUVECs in each group using the EdU method and found that HUVECs incubated in the LV‐uPAR‐RNAi‐CM group had the weakest proliferation activity compared to the LV‐GFP‐CM group (Figure 5C,D). The results of the tube formation experiment showed the same trend as the EDU experiment and the Transwell migration experiment (Figure 5E–I). In conclusion, we prove that hUC‐MSC sheets may promote angiogenesis by secreting uPAR to activate the PI3K/Akt pathway.

### 3.6 hUC‐MSC Sheets Transplantation to the Lesion Site Reduces the Cavity and Glial Scar and Promotes Angiogenesis

**FIGURE 6:**
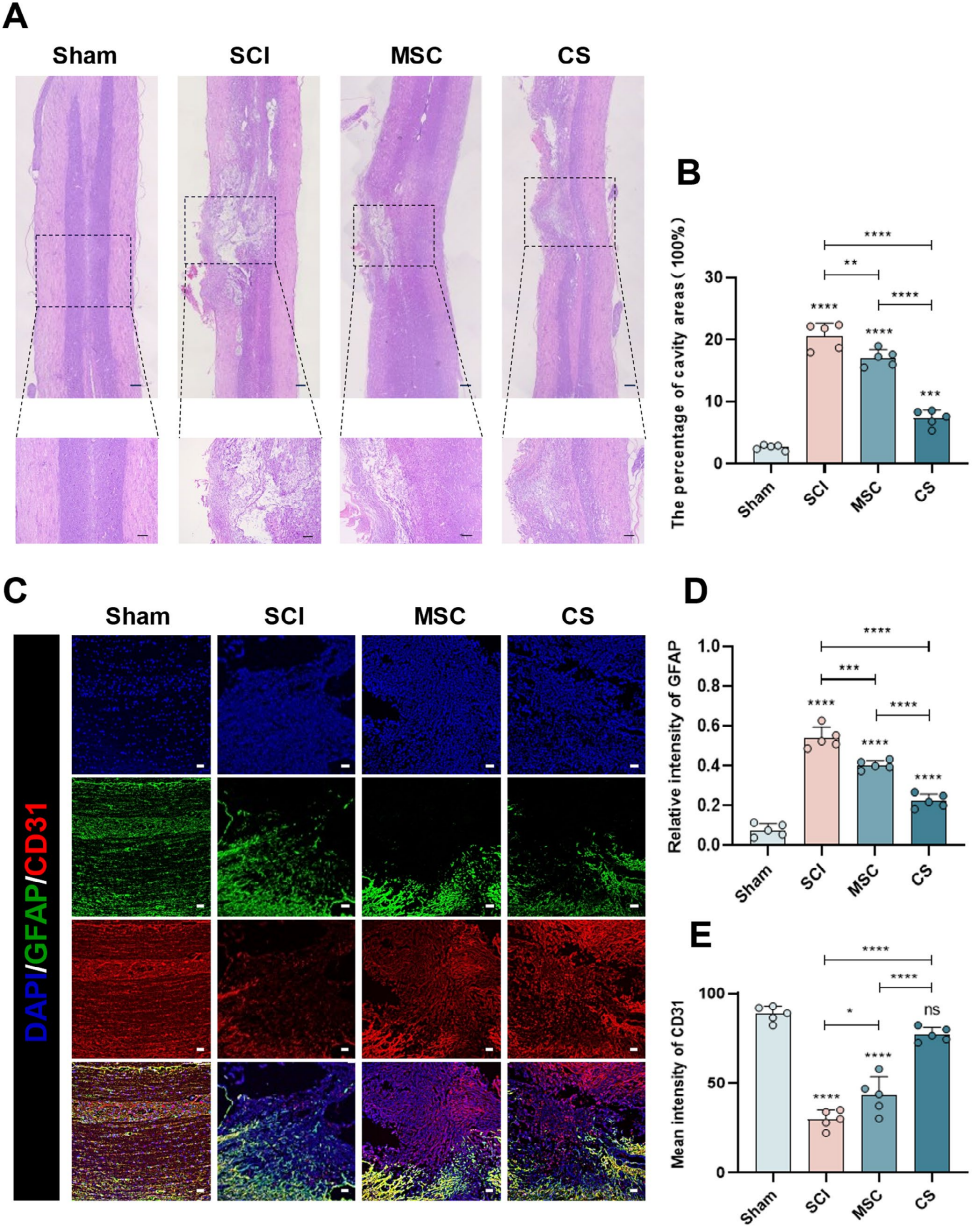
HE staining and immunofluorescence staining of GFAP and CD31 in spinal cord tissues of each group. (A) HE staining of spinal cord tissues. (B) Quantitative analysis of the cavity area in spinal cord tissues. (C) Detection of glial scar and vascular formation in regenerated spinal cord tissues (GFAP, green; CD31, red). (D, E) Quantitative analysis of GFAP and CD31 positive staining areas. Scale bar: 200 μm (A, B) and 50 μm (C). The data in panels (B, D, and E) passed the Shapiro–Wilk test and exhibit a Gaussian distribution. Data are presented as mean ± SEM (*n* = 5). *p < 0.05; **p < 0.01; ***p < 0.001; ****p < 0.0001; and ns, not significant.

Adult female SD rats were used as a model to evaluate the therapeutic effect of hUC‐MSC sheets on SCI. As shown (Figure 6A,B), HE staining showed the morphological changes of the spinal cord tissue. The Sham group was a normal spinal cord tissue. Fourweeks after surgery, an obvious atrophy occurred in the SCI group, and there was a large cavity in the injured area. In the MSC group, a cavity could still be observed, but continuous tissue appeared at the semi‐transected site. In the CS group, there was no obvious atrophy in the spinal cord tissue, and the new tissue around the damaged tissue increased significantly. Glial fibrillary acidic protein (GFAP) is one of the best biomarkers for glial scar formation after central nervous system injury [26], and glial scars often have an adverse effect on the recovery of neurological function [27]. Fourweeks after surgery, there was no significant difference in GFAP expression between the SCI group and the MSC group, and it was higher than that in the CS group (Figure 6C,D). The formation of blood vessels is crucial for maintaining the survival of cells around the damaged area. Since CD31 is a biomarker of HUVECs [28], the number of blood vessels after SCI can be quantified by CD31 immunofluorescence. We performed immunofluorescence staining of vascular marker CD31 (also known as platelet endothelial cell adhesion molecule‐1 (PECAM‐1)) on the spinal cord tissue. The results found that compared with the SCI group and the MSC group, the CS group showed more CD31 positive vascular structures with a wider distribution range. The expression of CD31 was the lowest in the SCI group, and the expression in the MSC group also increased, but the increase was not obvious (Figure 6C–E).

### 3.7 Promotion of the Regeneration of Neurons and Axons

**FIGURE 7:**
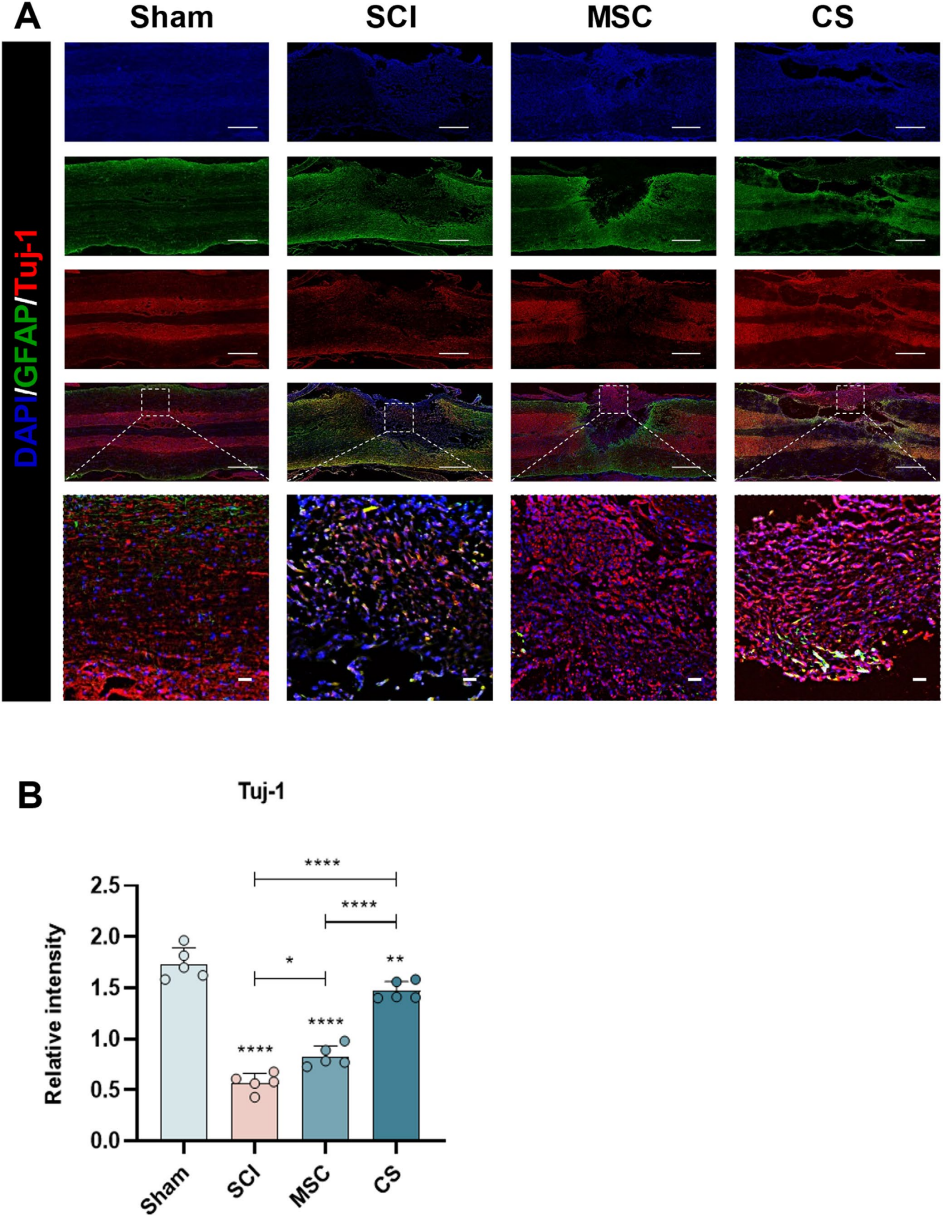
hUC‐MSC sheets implantation can promote nerve generation at the injury site. (A) Immunofluorescence images of neurons at the lesion site. Green IF represents the astrocyte marker GFAP. Red IF represents the neuronal marker Tuj‐1. (B) Statistical graph of the quantification of the relative fluorescence intensity of Tuj‐1. Scale bar: 1000 μm (A) and 50 μm (B). The data in panel (B) passed the Shapiro–Wilk test and exhibit a Gaussian distribution. Data are presented as mean ± SEM (*n* = 5). *p < 0.05; **p < 0.01; ***p < 0.001; ****p < 0.0001; and ns, not significant.

**FIGURE 8:**
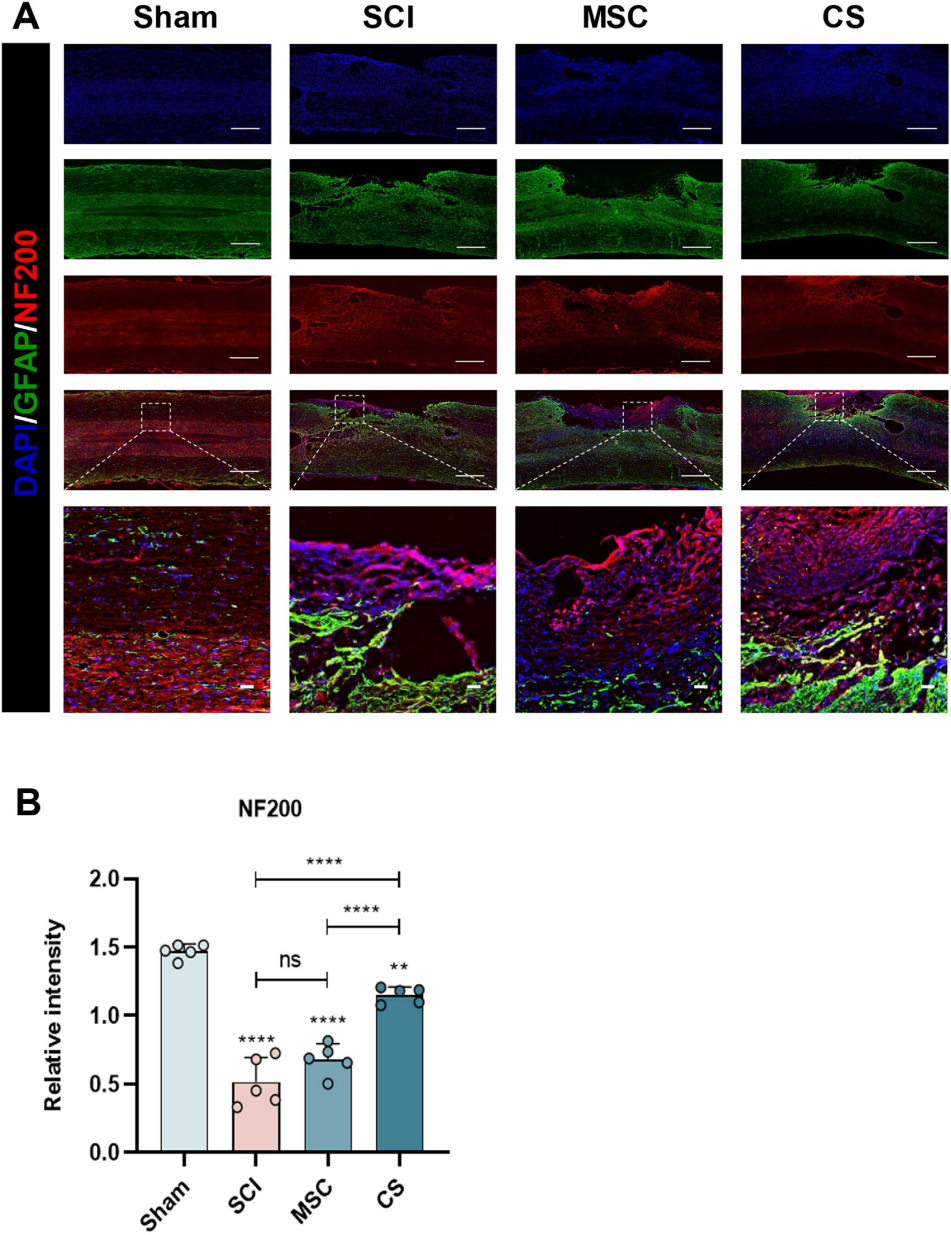
hUC‐MSC sheets implantation can promote axonal regeneration at the injury site. (A) IF imaging was used to evaluate axonal regeneration at the lesion site in each group. Green IF represents the astrocyte marker GFAP. Red IF represents the neuronal marker NF200. (B) Statistical graph of the quantification of the relative fluorescence intensity of NF200. Scale bar: 1000 μm (A) and 50 μm (B). The data in panel (B) passed the Shapiro–Wilk test and exhibit a Gaussian distribution. Data are presented as mean ± SEM (*n* = 5). *p < 0.05; **p < 0.01; ***p < 0.001; ****p < 0.0001; and ns, not significant.

To further determine whether the neural function of hUC‐MSCs or hUC‐MSC sheets can be improved on the premise of promoting histological changes and vascular repair in SCI, we used immunofluorescence to localize local neural progenitor cells and axons. Four weeks after injury, many Tuj‐1‐positive neurons were detected in the SCI center area of the CS group, while the neuronal regeneration rates in the SCI group and the MSC group were significantly lower (Figure 7A). By staining NF200‐positive axons at the injury site, it was found that the average fluorescence intensity of NF200 in the injury center of the CS group was significantly higher than that of other treatment groups, while only a small number of NF200‐positive axons were found in the SCI group and the MSC group (Figure 8A). It is worth noting that the statistical results showed that there was no significant difference in NF200 between the SCI and MSC groups and the SCI group (Figures 7B and 8B). The reason for this situation may be that after hUC‐MSCs are implanted into the injury site, they cannot stably stay in the injury center and play their role. As mentioned above, the CS group significantly promoted the regeneration of neurons and the growth and extension of axons.

### 3.8 Motor Function Recovery After SCI

**FIGURE 9:**
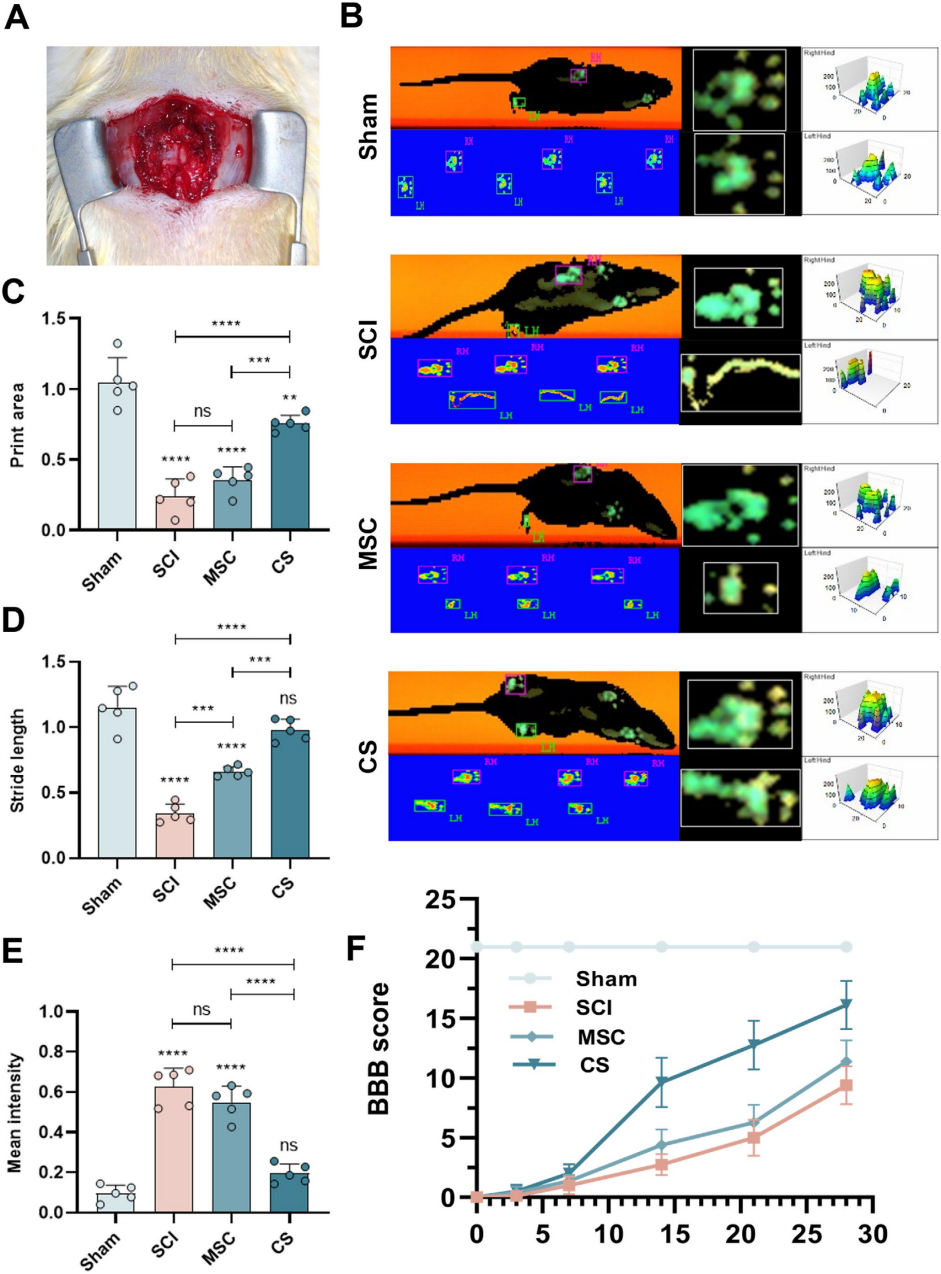
hUC‐MSC sheets promote the recovery of motor function in rats after SCI. (A) Schematic diagram of the rat left spinal cord hemisection injury model. (B) Representative footprints for analyzing the recovery of the hindlimb motor function. (C‐ E) Quantification of the print area, stride length, and mean intensity of the left hindlimb in each group at 4 weeks after surgery. (F) Measurement of the recovery of motor function in the left hindlimb by the BBB scale. The data in panel (B) passed the Shapiro–Wilk test and exhibit a Gaussian distribution. Data are presented as mean ± SEM (*n* = 5). *p < 0.05; **p < 0.01; ***p < 0.001; ****p < 0.0001; and ns, not significant.

To evaluate the therapeutic effect of hUC‐MSCs tablets on SCI, a 2‐mm injury was inflicted on the left side of the rat spinal cord during surgery and grafted cell sheets at the site of the defect (Figure 9A). The motor function recovery of rats could be visually assessed using footprint analysis (Figure 9B). Fourweeks after SCI, rats in the SCI group showed only dragging and crawling of the left hind limb. Rats in the MSC group showed coordinated movements of the forelimbs and hind limbs, but the gait trajectories of the left hind limb were not completely consistent. In particular, the paw print area of the CS group was very close to that of the Sham group, and more coordinated front and hind limb movements were observed. Data analysis showed that no statistically significant differences were observed between the experimental and Sham groups in terms of the stride length and average strength (Figure 9C–E), suggesting that the administration of therapeutic hUC‐MSCs sheets enhanced the motor function of rats.

The BBB score was used to assess the recovery of the motor function in rats after surgery (Figure 9F). Before surgery, each rat had normal motor function of the left hind limb (21 points). After the injury caused by surgery, the rats exhibited complete paralysis of the left hind limb (0 points). In terms of postoperative recovery, no significant difference was observed between the CS and the SCI and MSC groups at 1 week after surgery, whereas by week 2, the scores of neither the SCI nor the MSC group exceeded 7, which indicated that the rats had limited ability to repair the tissues after the injury. The CS group showed a better recovery of motor function compared to the SCI and MSC groups, but the effect was not significant. At 4 weeks after surgery, the statistically significant difference observed in the MSC group compared to the SCI group remained insignificant. However, most of the rats in the CS group showed sustained hand weight‐bearing movements and coordinated anterior and posterior limb movements with scores ranging from 15 to 18. Similarly, the majority of rats in the MSC group exhibited supported palm weight‐bearing movements as well as coordinated anterior–posterior limb movements, with scores ranging from 11 to 13 points. Rats in the SCI exhibited only ankle movements (6–8 points). We found that the results of the BBB scores coincided with the results of the footprint analysis, both supporting the idea that hUC‐MSCs sheets accelerate functional recovery after SCI.

**FIGURE 10:**
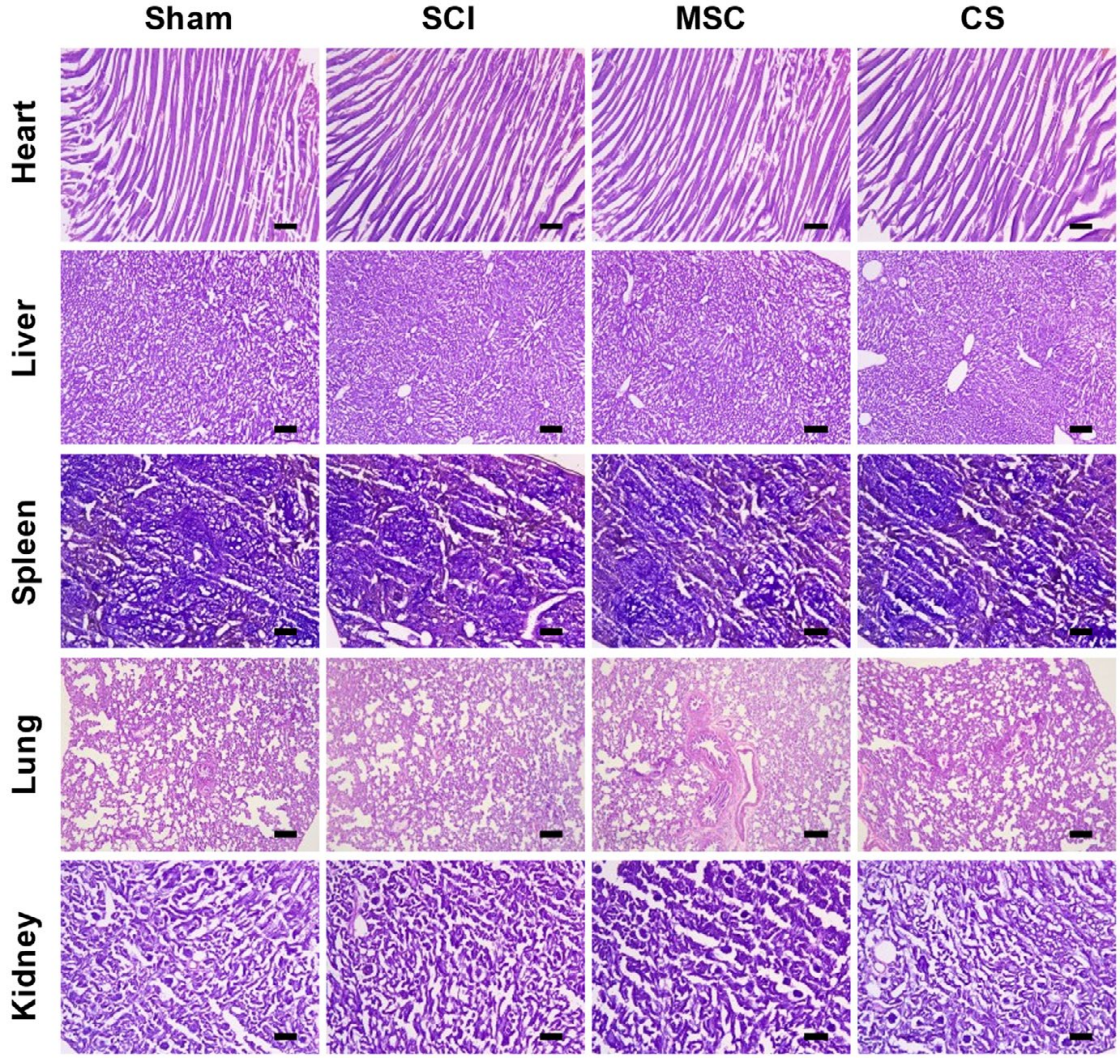
hUC‐MSC sheets implantation shows no toxicity in vivo. H&E staining of the heart, liver, spleen, lung, and kidney in each group of rats. Scale bar: 200 μm.

The objective of this study is to assess the safety of hUC‐MSC sheets, and various organs were stained with H&E. The results are shown in Figure 10. hUC‐MSC sheets are biocompatible and have no significant impact on general tissues. Pathological examinations of the heart, liver, spleen, lung, and kidney in all treatment groups yielded essentially identical results, with no significant abnormalities identified. This evidence substantiates the hypothesis.

## 4 Discussion

Limited functional recovery following SCI can be attributed, at least in part, to two factors: the poor reparability of neurons and the secondary deterioration of the microenvironment [29]. The improvement of the localized blood supply is related to the changes that occur in the microenvironment of the injury area. The initial mechanical injury results in the disruption of the microvascular integrity and the breakdown of the BSCB in adjacent tissues, which in turn precipitates ischemia and hypoxia. This cascade of events gives rise to pathological alterations, including neural tissue necrosis and the infiltration of inflammatory cells [30]. Complete vascular results and abundant blood supply can remove local apoptotic cell debris and metabolites and promote the delivery of oxygen/nutrients [31]. It can be reasonably deduced that the vascular system of the spinal cord plays a pivotal role in the process of SCI and subsequent repair. Otherwise, if the neural tissue does not receive an appropriate supply of nutrients from the blood [32, 33], its repair may be affected. In addition, vascular endothelial cells, as the most important component of the vascular system, experience a significant loss of endothelial cells in acute SCI. Therefore, in the treatment of patients with SCI, it should be considered that the generation of endothelial cells should be promoted as much as possible to assist in blood vessel reconstruction and create a microenvironment conducive to neural repair. The use of mesenchymal stem cells for treatment is currently considered an effective strategy to promote vascular repair in SCI, often through intramedullary injection or intravenous injection to implant cells [9], but the therapeutic effect is limited. In order to better exert the role of cells, we combined cell sheet technology to prepare hUC‐MSC sheets and used this method to promote early spinal cord angiogenesis.

In this study, we used hUC‐MSCs as the cell source because hUC‐MSCs can secrete a large number of pro‐angiogenic factors, which have been widely used in many ischemic diseases [34]. Past studies have confirmed the effectiveness of using hUC‐MSC sheets therapy [35, 36]. It is worth noticing that the formation of cell sheets not only helps cells stay locally in the injury for a long time but also enhances the paracrine effect of cells due to the presence of the extracellular matrix (ECM) [37], which is consistent with the conclusion we obtained during the experiment. In addition, the ECM provides a natural growth environment for cells, connects the two ends of the SCI, effectively fills the defect, and acts as a scaffold. Implanting hUC‐MSCs sheets into the SCI site of rats not only locally gathers the cells together but also fills the spinal cord space, supplements the ECM, and promotes nerve regeneration [38]. H&E staining showed that there were different degrees of cavities in the SCI group and the MSC group, notable tissue proliferation and structural dysfunction were observed at the injury site, with evidence suggesting an expansion of the injury area. In contrast, the tissue arrangement in the CS group was more orderly at the injury site, and the repair degree was significantly higher than that of the SCI group and the MSC group, with the largest difference from the SCI group. In addition, after SCI, a large number of glial scars formed by GFAP‐positive astrocytes will form at the local injury, which will seriously affect the growth of neurons and axon extension [27, 37]. In previous studies, BMSC sheets and ADSC sheets could effectively inhibit the formation of glial scars [13, 39]. We used GFAP fluorescence staining to label the astrocytes at the local injury, and the results confirmed that hUC‐MSC sheets could also achieve a similar effect. We found obvious cell proliferation in the MSC group and the CS group during the experiment. To verify whether the new cells in the injury area are neurons and axons, we used Tuj‐1 and NF200 immunofluorescence to mark the local injury and found that there was obvious neuron regeneration and axon extension in the CS group. Nevertheless, the reparative impact of the SCI and MSC groups was constrained, and no notable discrepancy was observed between the two groups. This situation may be caused by the serious cell loss in the MSC group, which cannot effectively play the role of stem cells.

We used MSC‐CM and CS‐CM to observe their indirect effects on the proliferation of vascular endothelial cells. The findings demonstrated that both MSC‐CM and CS‐CM were capable of promoting the proliferation and migration of endothelial cells, as well as facilitating the formation of a two‐dimensional structure with capillary‐like lumens. Furthermore, the fluorescence labeling of CD31 in the spinal cord tissue revealed that the mean fluorescence intensity was significantly greater in the CS group than in the MSC group and the SCI group, indicating that CS can effectively promote the proliferation of vascular endothelial cells and replenish the tissue catastrophe at the injury site. Prior research has demonstrated that distinct subtypes of endothelial cells are involved in neovascularization processes. Some of these cells are primarily responsible for guiding the migratory process, while others are responsible for the regulation of proliferation. For instance, endothelial “tip cells” that spearhead the sprouting of new blood vessels rely predominantly on the projection of filopodia, which are instrumental in both migration and guidance. “Stem cells” are able to track behind such extensions and facilitate the elongation of existing branches. Furthermore, they are capable of proliferating under the direction of tip cells. It is hypothesized that tip cells facilitate the initiation of sprouting at the damaged ends of blood vessels, displaying solely migratory potential, but no proliferative capacity [40, 41]. Stem cells differentiate into tip cells, which form the body of the growing bud. After spatial rearrangement, a lumen can be established, enabling the flow of blood. Consequently, the proliferation and migratory capacity of endothelial cells can be elucidated through methodologies such as CCK‐8 array and wound healing assay. This can contribute to the comprehension of the angiogenic pattern in patients with SCI and provide guidance for clinical management of such injuries. It is thus imperative that future investigations focus on elucidating the precise structural pattern changes associated with angiogenesis following SCI, with the aim of establishing a clearer understanding of the underlying mechanisms governing the progressive relationship between cell migration and proliferation.

In order to ascertain which key pro‐angiogenic chemokines and/or growth factors hUC‐MSCs and hUC‐MSC sheets are capable of secreting, a combination reaction of cell membranes (preloaded with growth factor binding targets) and MSC‐CM and CS‐CM was employed, resulting in the identification of high expressions of UPAR, MMP‐1, GSF‐1, and VEGF. These pro‐angiogenic factors are of great importance in the development of new blood vessels and subsequent physiological homeostasis. Activation of the PI3K/Akt pathway has been demonstrated to elicit a multitude of fundamental cellular responses intrinsic to angiogenesis, including survival, migration, and the formation of tubes. In light of the array screening results, the aim was to confirm whether this pathway is involved in the promotion of survival, migration, and tube formation of endothelial cells by hUC‐MSCs and hUC‐MSC sheets. It was established that this pathway is involved by measuring the phosphorylation levels of PI3K and Akt. Nevertheless, it is of paramount importance to ascertain the specific role of a given factor among the pro‐angiogenic factors present in MSC‐CM and CS‐CM. Therefore, combining the array screening and the protein kinase phosphorylation trend in Western blot, we prove that uPAR may be a key player in activating the PI3K/Akt pathway. Current studies suggest that uPAR was originally thought to contribute to the directed invasion of migratory cells, but it is now increasingly evident that this protease receptor triggers a large number of cellular responses, including cell adhesion, differentiation, proliferation, and migration in a non‐proteolytic manner and promotes vascular development and angiogenesis stimulation [42, 43]. In subsequent validations, we chose to use lentiviral transfection to successfully interfere with uPAR in hUC‐MSCs.

We constructed hUC‐MSC sheets capable of reducing uPAR secretion. Subsequently, we used LV‐CM and LV‐uPAR‐RNAi‐CM to culture HUVECs, analyzed the results obtained from Western blot, and proved that uPAR indeed promotes the proliferation, migration, and tube formation of endothelial cells by activating the PI3K/Akt pathway. The results of subsequent HUVECs biological behavior experiments also prove this. It is noteworthy that the phosphorylation levels of PI3K and AKT kinases in HUVECs in the LV‐uPAR‐RNAi‐CM group remained higher than those in the control group throughout the study. This indicates that, in addition to uPAR, the cytokines secreted by hUC‐MSCs also promote the proliferation and migration of endothelial cells by activating the PI3K/AKT pathway. Interestingly, in our screened pro‐angiogenic factor array, VEGF is also one of the highly secreted cytokines of hUC‐MSCs and hUC‐MSC sheets, and VEGF has been widely studied and believed to be able to activate the PI3K/AKT pathway to promote the proliferation and migration of endothelial cells [41, 44, 45]. Therefore, VEGF may be one of the key factors for hUC‐MSCs and hUC‐MSC sheets to exert pro‐angiogenic effects. Additionally, the classical PI3K/Akt pathway participates in multiple processes and reactions. With regard to neurons, a number of studies have demonstrated that the PI3K/Akt signaling pathway plays a pivotal role in neuroprotection, axonal regeneration, and neurogenesis [46, 47], and the activation of the PI3K/AKT signaling pathway has been observed to inhibit the inflammatory response and apoptosis in the subacute phase of SCI [48].

## 5 Conclusions

**FIGURE 11:**
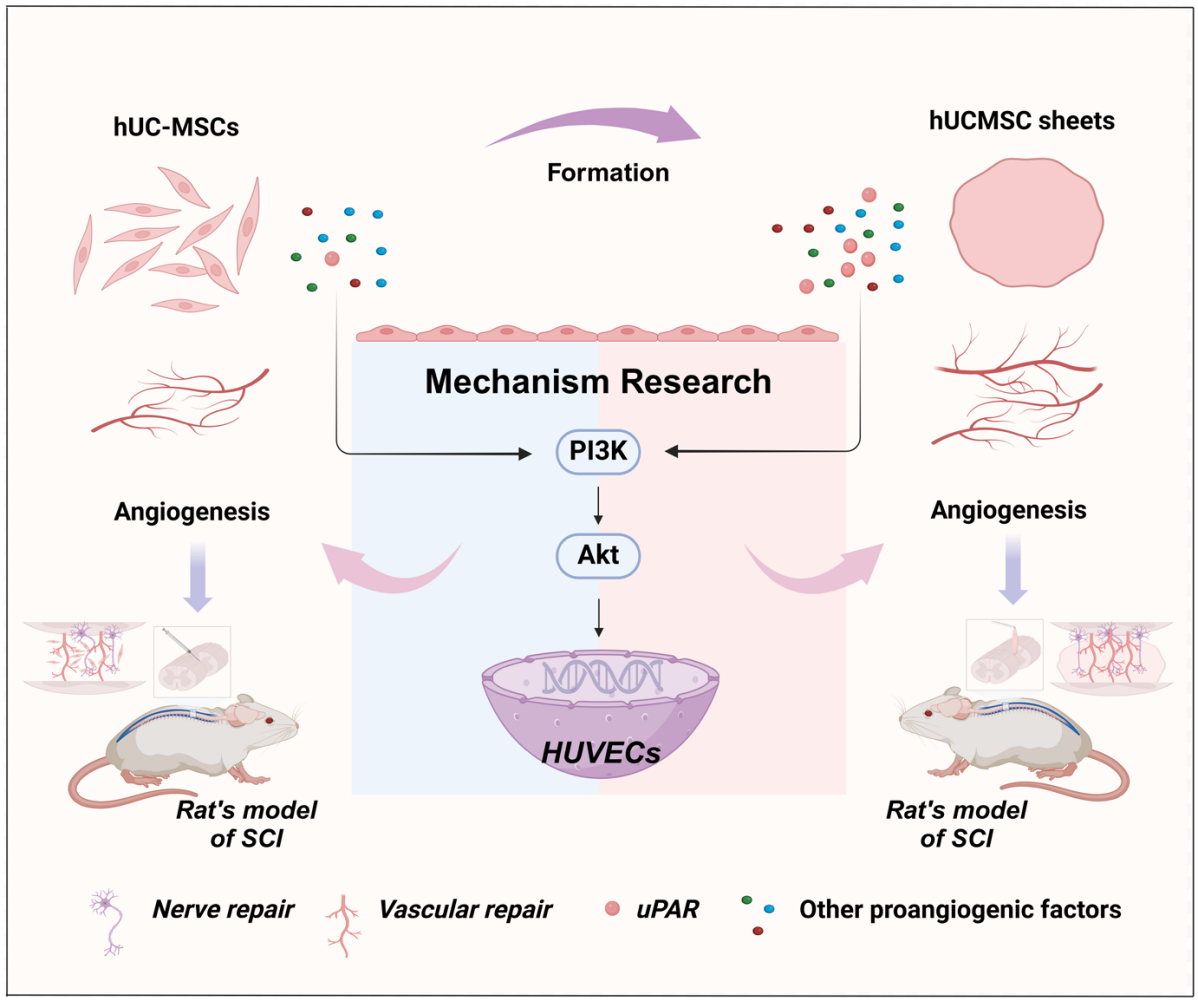
Enhanced therapeutic efficacy of human umbilical cord mesenchymal stem cells in spinal cord injury by the formation of cell sheets and its mechanism elucidation. Created with BioRender.com.

In conclusion, the mechanisms by which hUC‐MSCs promote SCI repair are multifactorial, and our current study attempts to elucidate and clarify some of these mechanisms regarding pro‐angiogenesis (Figure 11). hUC‐MSC sheets retain and enhance the biological functions of hUC‐MSCs, compensating for the disadvantages in stem cell transplantation methods, which is consistent with the original design of our research strategy. The fundamental premise of sequential stem cell transplantation therapy is to facilitate the repair and supplementation of an unfavorable microenvironment in the aftermath of a SCI. hUC‐MSC sheets perfectly undertake this task, effectively inhibiting glial scarring, promoting neuronal regeneration and axon extension, and angiogenesis, injecting fresh ideas into the research of cell therapy for SCI. Our elucidation of the mechanisms further provides a basis for the efficacy of its treatment.

## Author Contributions

Xiaoqing Chen and Yulin Zhao conceived and designed the study. Xiaoqing Chen, Yulin Zhao, Zhengchao Wu, Yuchen Zhou, Cheng Chen, Yang Lu, Heng Wang, Tao Xu, and Changwei Yang performed the experiments. Yulin Zhao and Zhengchao Wu analyzed the data. Yulin Zhao and Yuchen Zhou performed the mechanism drawing. Yulin Zhao and Xiaoqing Chen wrote the manuscript. All authors reviewed and approved the final manuscript.

## Conflicts of Interest

The authors declare no conflicts of interest.

## Supporting information


Data S1.

## Data Availability

The data that support the findings of this study are available from the corresponding author upon reasonable request.
