# Supplementary figures and images for: Cell Sheets Formation Enhances Therapeutic Effects of Human Umbilical Cord Mesenchymal Stem Cells on Spinal Cord Injury
Source: CNS Neurosci Ther. 2024 Dec 13;30(12):e70163. doi: 10.1111/cns.70163 (PMC11638885; doi:10.1111/cns.70163)

**Raw Data of WB bands:**

**Figure4.**

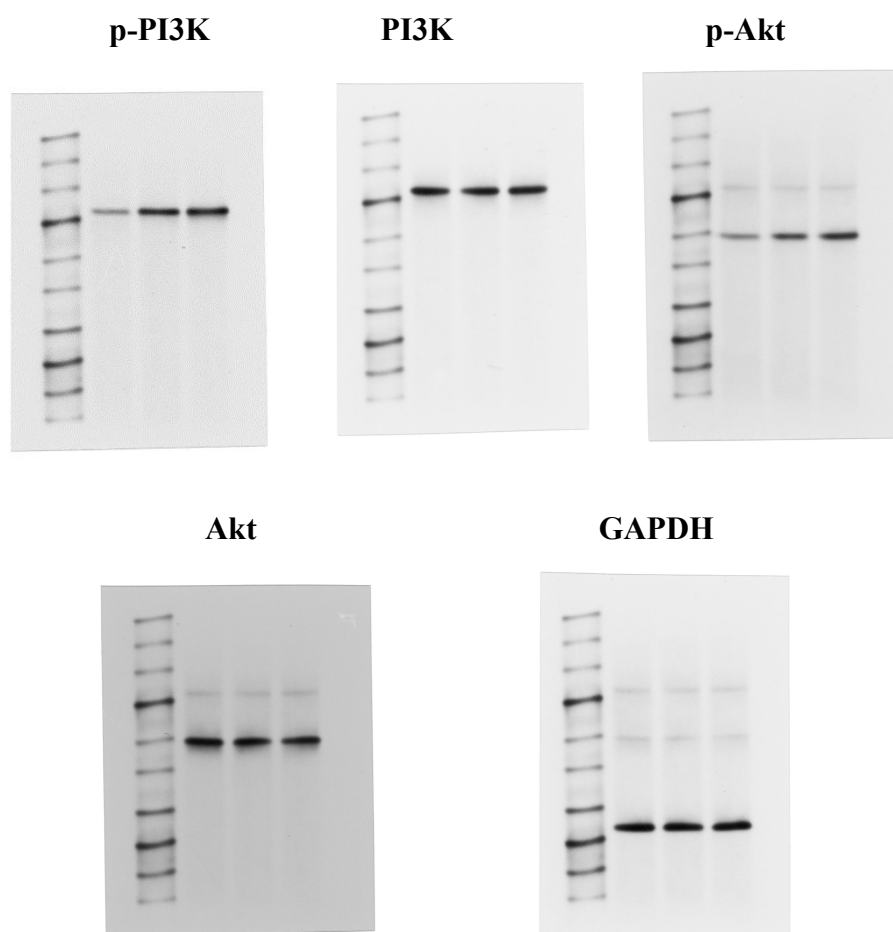

**Figure5.**

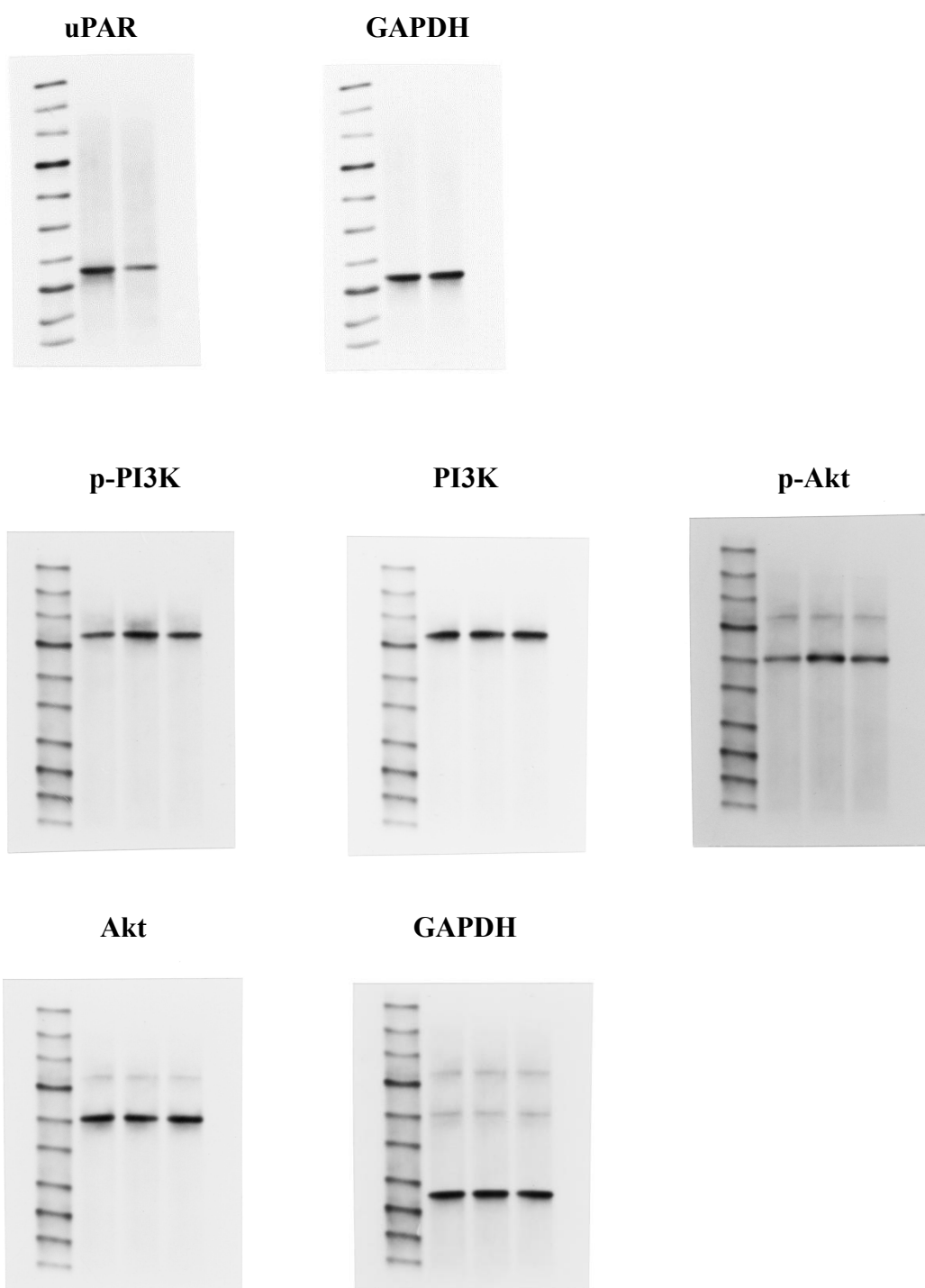

Supplement: Supplementary file 1 — Data S1. [file CNS-30-e70163-s001.pdf]
